# Supplementary material for: Proton tautomerism for strong polarization switching
Source: Nat Commun. 2017 Feb 16;8:14426. doi: 10.1038/ncomms14426 (PMC5316872; doi:10.1038/ncomms14426)
Supplement: Supplementary Information — Supplementary Figures, Supplementary Tables, Supplementary Note and Supplementary Reference [file ncomms14426-s1.pdf]

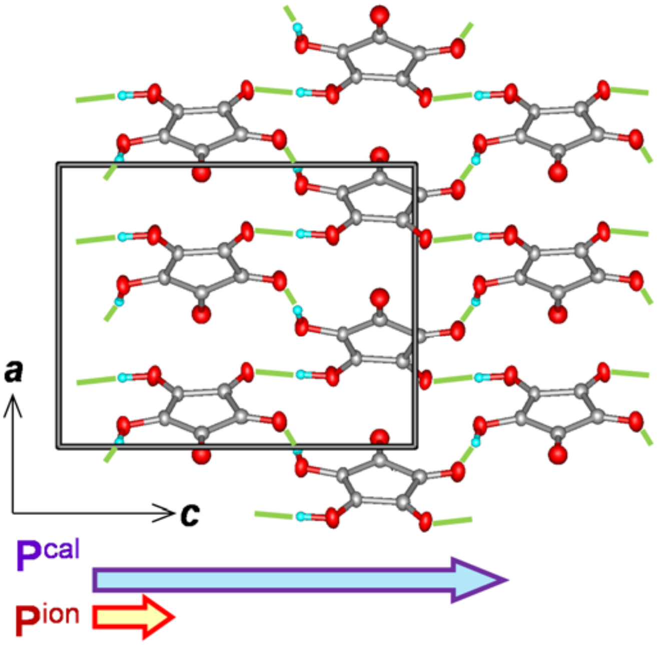

Supplementary Figure 1: Crystal structure of CRCA viewed along the crystallographic *b*-direction. Open arrows compare the direction and relative amplitudes of the (total) theoretical polarization vector $\mathbf{P}^{cal}$ (in agreement with the experimentally optimized polarization vector $\mathbf{P}^{exp}$) with those estimated from the proton-displacement (point-charge) model $\mathbf{P}^{ion}$. Green solid lines represent the intermolecular hydrogen bonds.

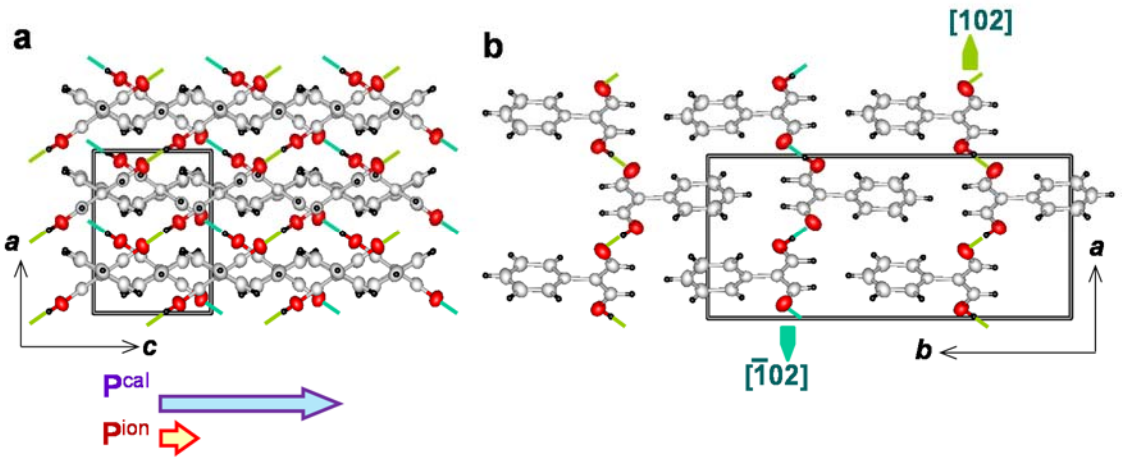

Supplementary Figure 2: Crystal structures of PhMDA. Molecular arrangement viewed along the (**a**) crystallographic *b*- and (**b**) *c*-directions. Open arrows in (a) compare the direction and relative amplitudes of the total polarization ($\mathbf{P}^{cal}$ in agreement with $\mathbf{P}^{exp}$) and the estimate of the proton-displacement polarization $\mathbf{P}^{ion}$. Green solid lines denote the intermolecular hydrogen bonds.

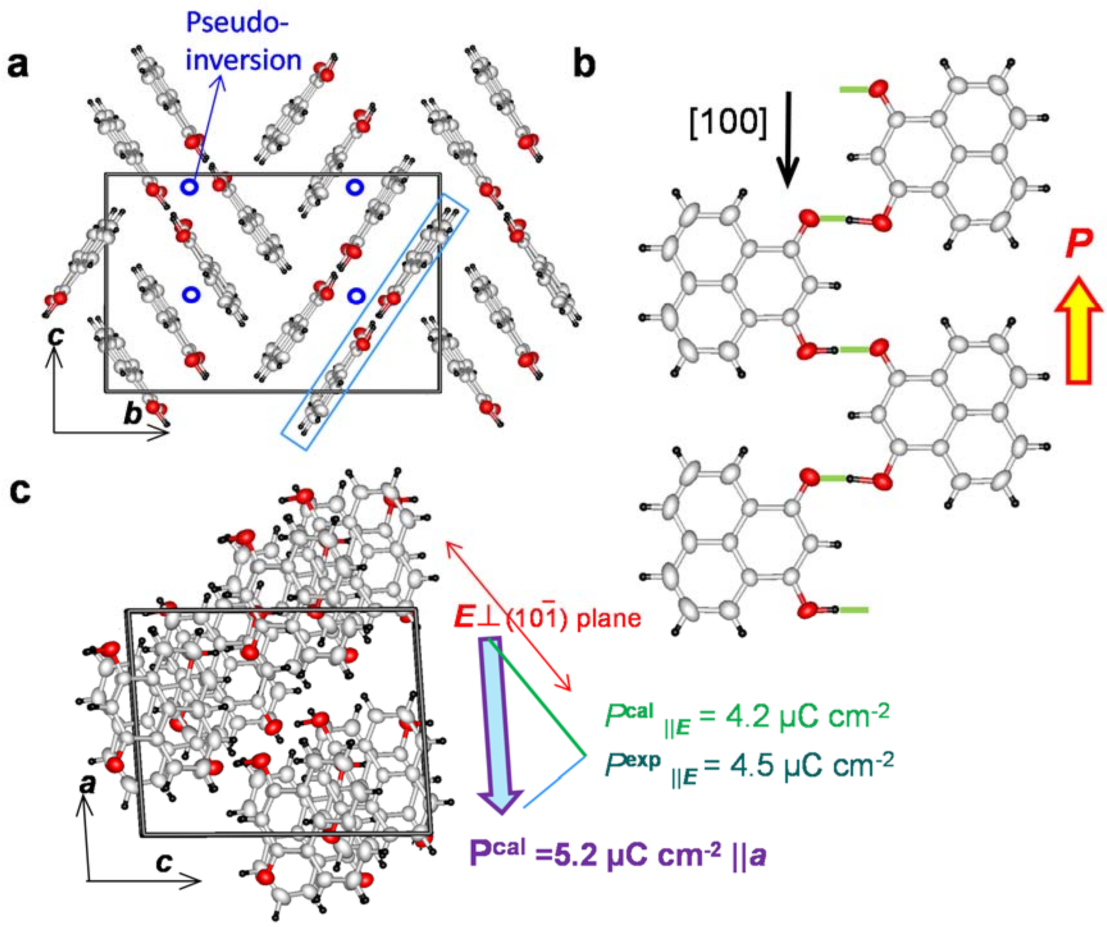

Supplementary Figure 3: Crystal structure of α-form HPLN. (**a**) Molecular arrangement viewed along a hydrogen-bonded chain (thin box), which is parallel to the crystallographic *a*-direction. (**b**) Direction of the spontaneous polarization along the chain of hydrogen bonds (pale green lines). (**c**) Molecular arrangement viewed along the *b*-direction and the calculated polarization vector $\mathbf{P}^{cal}$ (open arrow). Note that the experimental polarization $\mathbf{P}^{exp}$ measured normal to the crystal (10$\bar{1}$) plane is comparable to the electric field (*E*)-direction component of $\mathbf{P}^{cal}$.

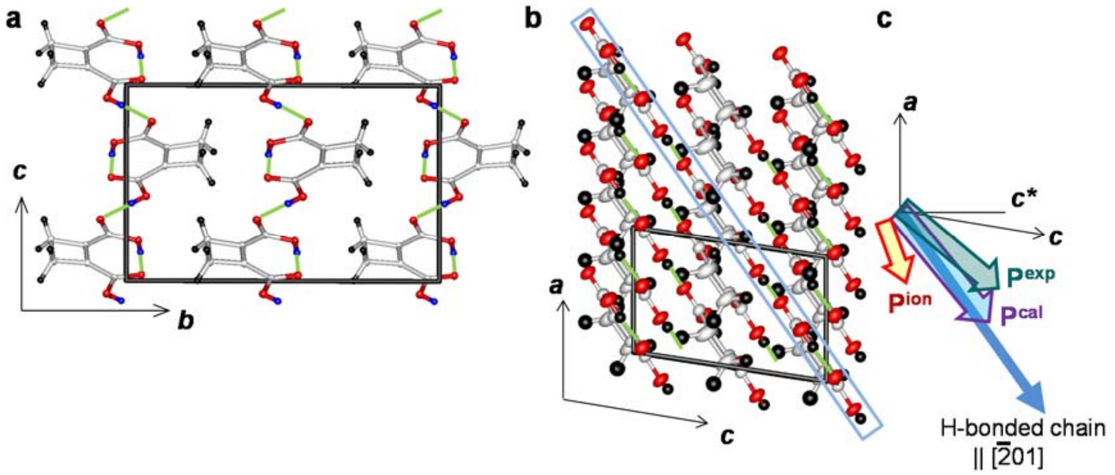

Supplementary Figure 4: Crystal structure of CBDC. (**a**,**b**) Molecular arrangement viewed along the crystallographic *a*- and *b*-directions. Solid green lines denote OH···O hydrogen bonds. (**c**) Direction and relative amplitude of the theoretical $\mathbf{P}^{cal}$, experimentally optimized $\mathbf{P}^{exp}$, and estimate of the proton-displacement polarization $\mathbf{P}^{ion}$ with correspondence to the crystallographic directions. Note that the sign of each component of $\mathbf{P}^{exp}$, which could not be identified by the *P–E* hysteresis experiments alone, is assumed to be the same as that of $\mathbf{P}^{cal}$.

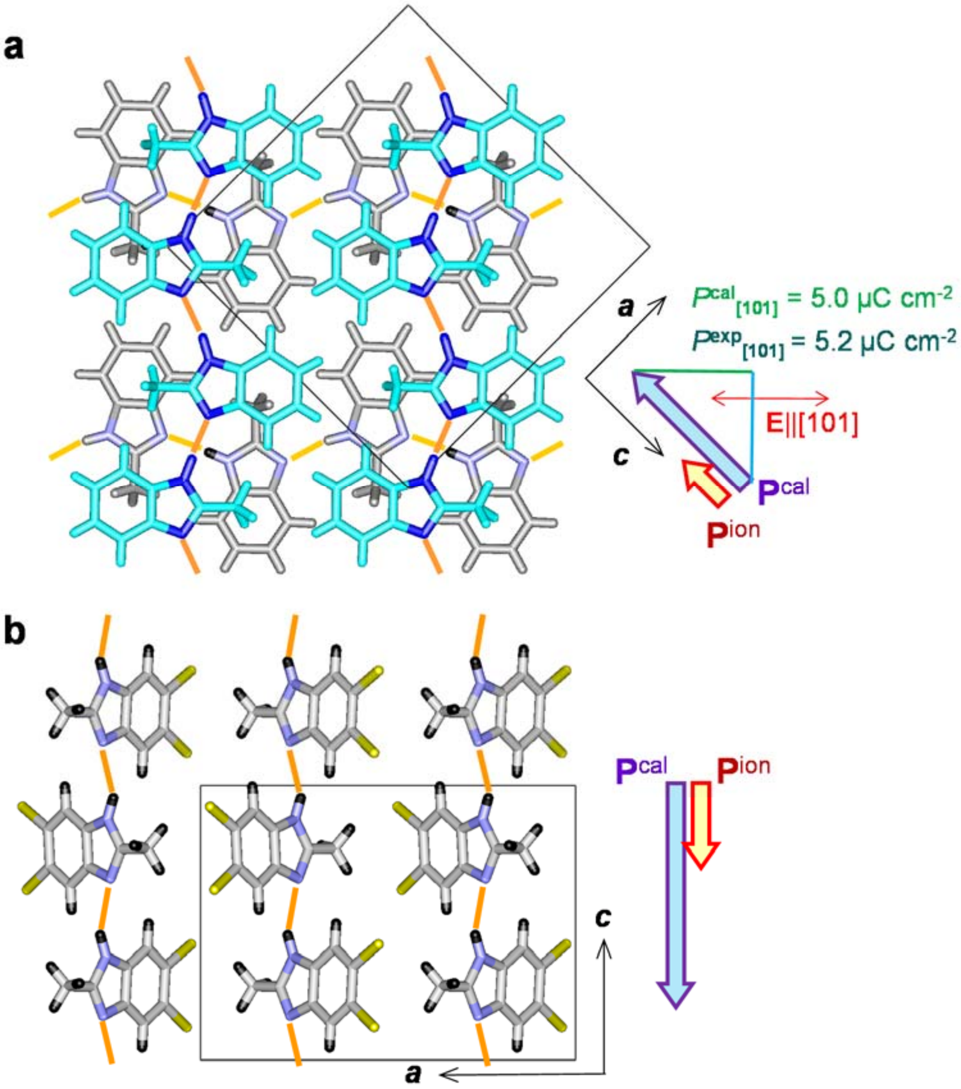

Supplementary Figure 5: Crystal structure of benzimidazoles. (**a**) MBI and (**b**) DC-MBI crystals viewed along the crystallographic *b*-direction. Solid orange lines denote intermolecular hydrogen bonds. Open arrows represent the direction and relative amplitude of the theoretical $\mathbf{P}^{cal}$ and experimentally optimized $\mathbf{P}^{exp}$ polarizations.

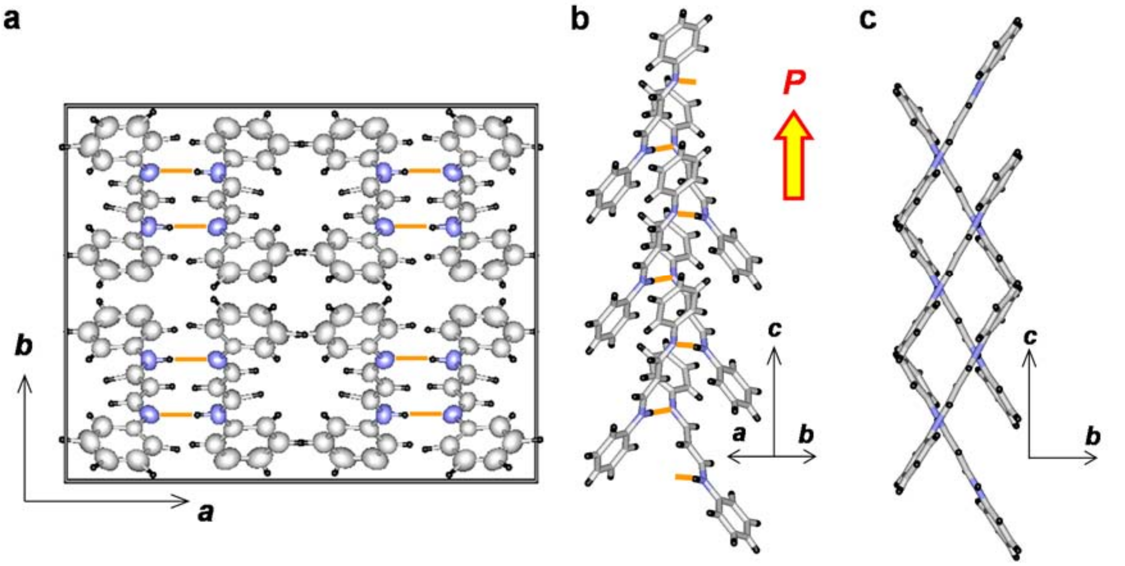

Supplementary Figure 6: Crystal structure of ALAA. (**a**) Molecular packing viewed along the crystallographic *c*-direction. The extracted hydrogen-bonded chains and polarization (open arrow) viewed along the (**b**) [101] and (**c**) [100] directions. Solid orange lines represent intermolecular hydrogen bonds.

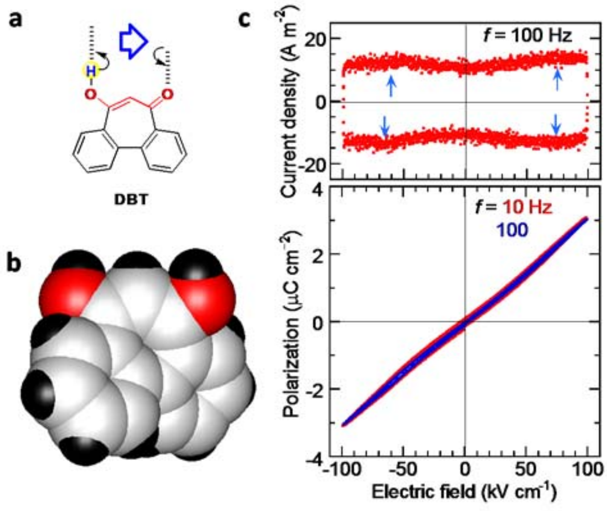

Supplementary Figure 7: Properties of a DBT crystal. (**a**) Chemical structure. The chain polarity (open arrows) can be inverted by the proton transfer (round arrow) over a hydrogen bond (thick broken line). (**b**) Proton-disordered molecular structure with twofold symmetry at room temperature. (**c**) The *c*-direction polarization (*P*) versus electric field (*E*) hysteresis loops (bottom) and corresponding current density (*J*) versus electric field (*E*) curves at 100 Hz (top). Arrows indicate the (switching) peaks in the current.

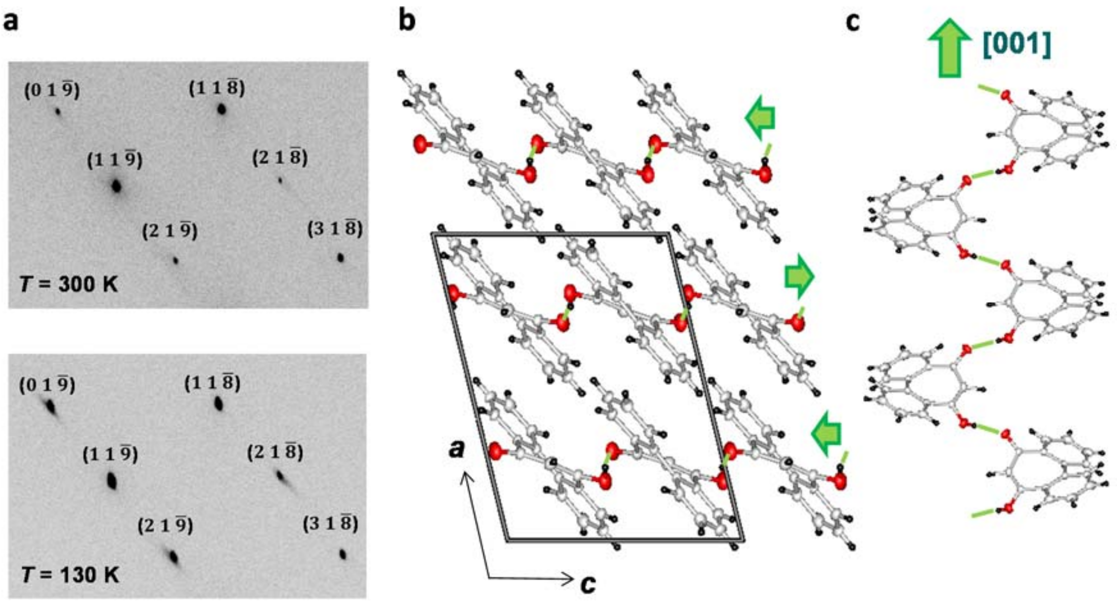

Supplementary Figure 8: Structural properties of a DBT crystal. (**a**) Synchrotron X-ray photographs at T = 300 K (top) and 130 K (bottom). (**b**) Molecular packing and direction of the chain dipole viewed (open arrow) along the crystallographic *b*-direction. (**c**) The extracted hydrogen-bonded chain viewed in the [100] direction. Solid green lines denote OH···O hydrogen bonds.

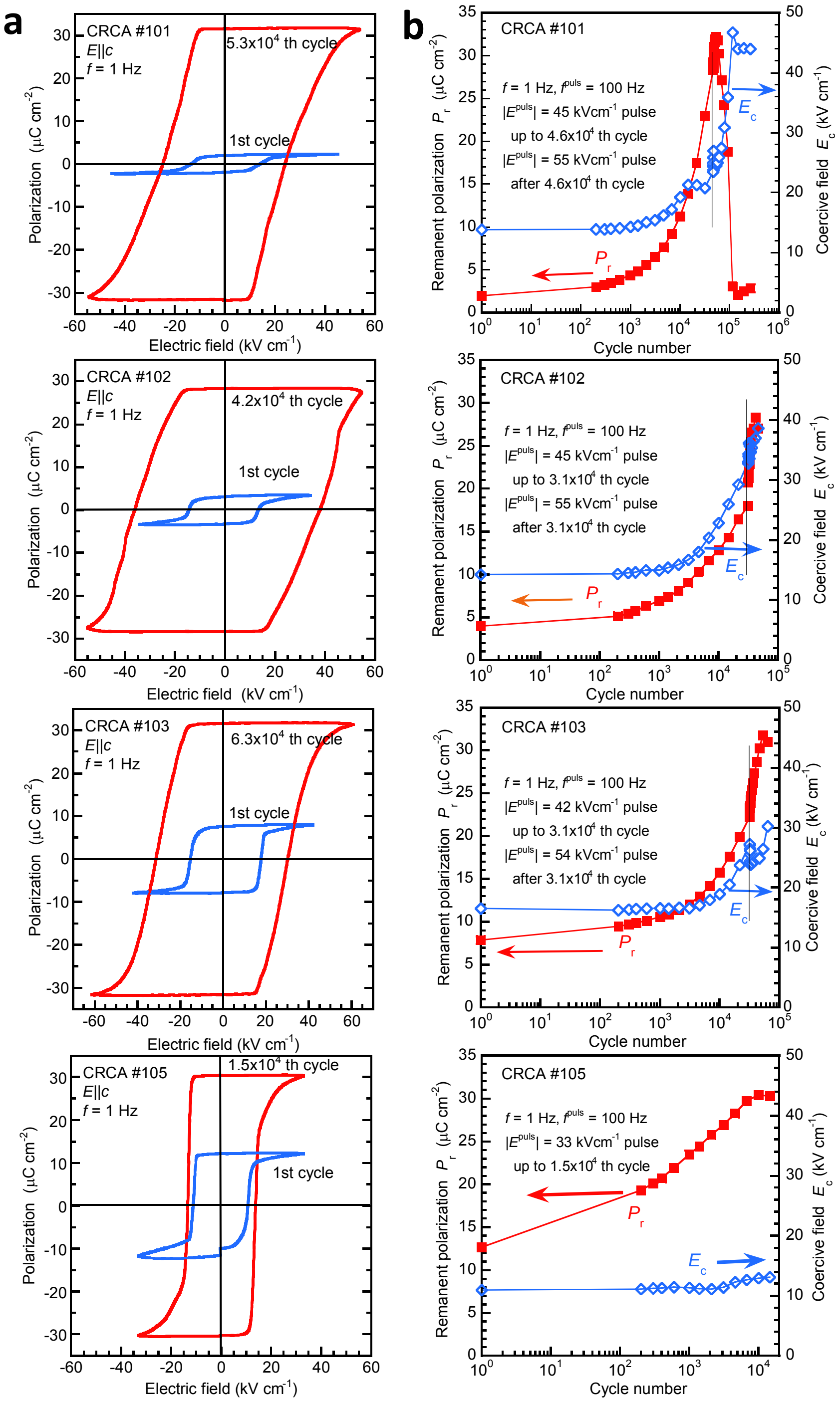

Supplementary Figure 9: Optimization of the electric polarization (*P*) versus electric field (*E*) hysteresis loops of four CRCA crystal specimens. (**a**) Comparison of *c*-direction polarization loops before and after optimization. (**b**) Evolution of remanent polarization P_r (red squares) and coercive field E_c (blue diamonds) in an endurance measurement mode. A continuous rectangular-pulse voltage (f = 100 Hz) for the compulsory switching, and the triangular waveform voltage (f = 1 Hz) for the *P*–*E*

hysteresis measurements were applied. See the inset for a schematic waveform in an endurance measurement mode. The hysteresis measurements were repeated with a constant interval in a logarithmic time scale.

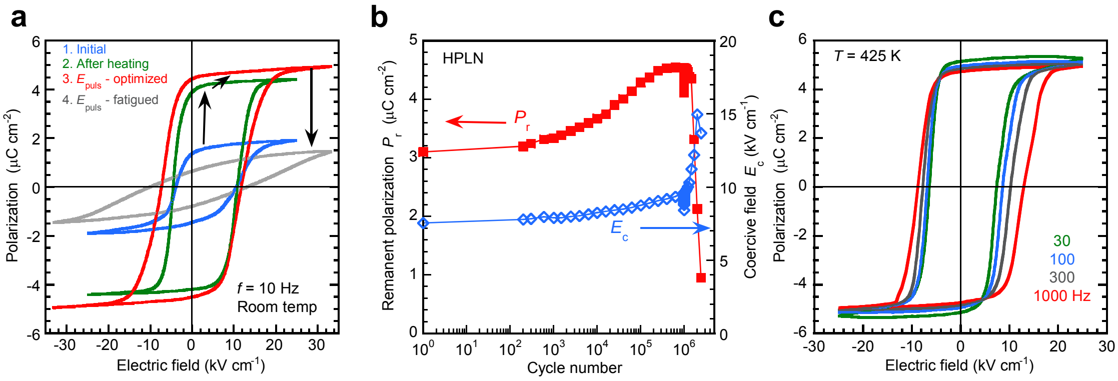

Supplementary Figure 10: Optimization of the *P–E* hysteresis loops for polarization normal to the $(10\bar{1})$ plane of the α-form HPLN. (**a**) *P–E* hysteresis loops at f = 10 Hz with the history from thermal and electric optimization to fatigue. (**b**) Evolution of remanent polarization P_r (red squares) and coercive field E_c (blue diamonds) in an endurance measurement mode. A continuous rectangular-pulse voltage (f = 100 Hz, E^{puls} = 33 kV cm^{-1}) for the compulsory switching and the triangular waveform voltage (f = 10 Hz, E^{max} = 33 kV cm^{-1}) for the *P–E* hysteresis measurements were applied. (**c**) *P–E* hysteresis loops at various frequencies at the highest temperature examined (T = 425 K).

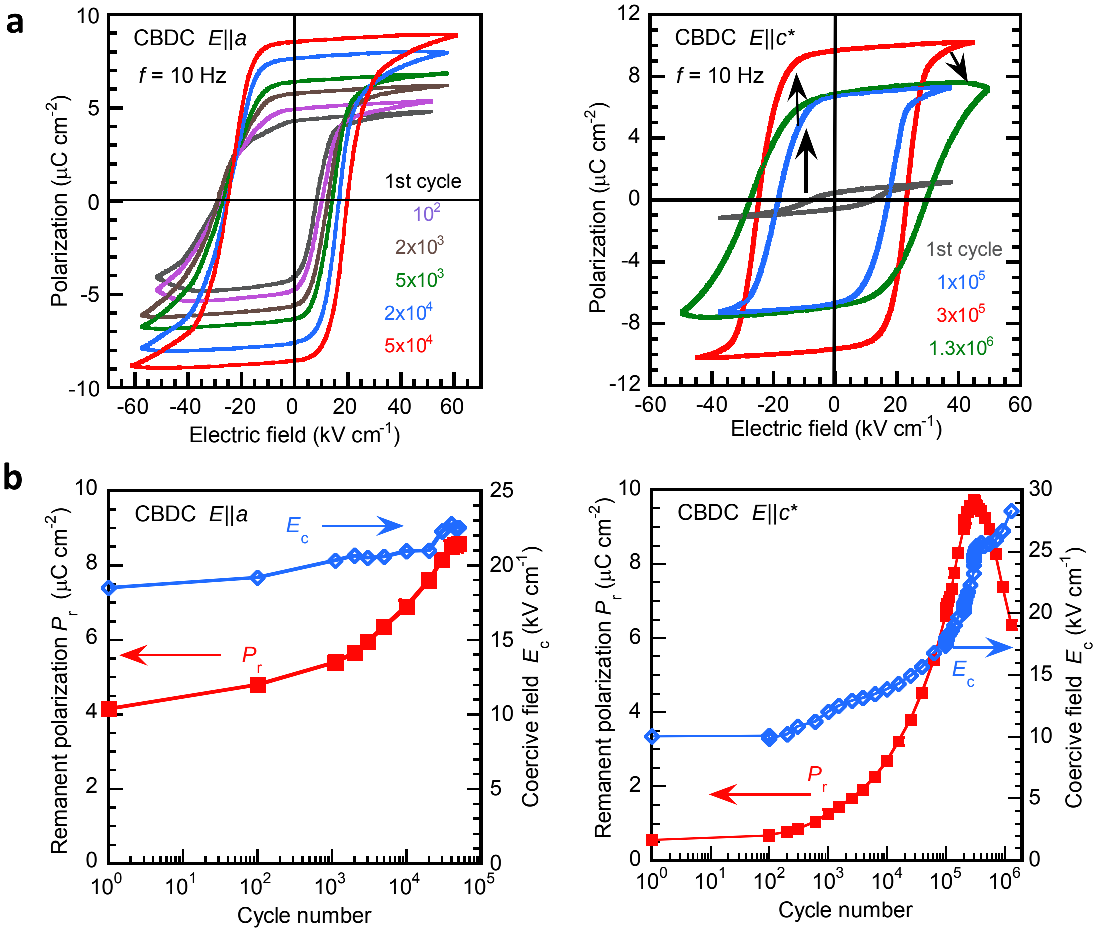

Supplementary Figure 11: Optimization of the *P*–*E* hysteresis loops of *a*- (left) and *c-direction polarizations (right) of CBDC crystals.** (**a**) Comparison of the *P*–*E* hysteresis loops before and after optimization. (**b**) Evolution of P_r (red squares) and E_c (blue diamonds) in an endurance measurement mode. A continuous triangular waveform voltage (f = 10 Hz, E^{max} = 60 kV cm^{-1} for $E||a$) or rectangular-pulse voltage (f = 100 Hz, E^{puls} = 45 kV cm^{-1} for $E||c^*$) for the compulsory switching and the triangular waveform voltage (f = 10 Hz) for the *P*–*E* hysteresis measurements were applied.

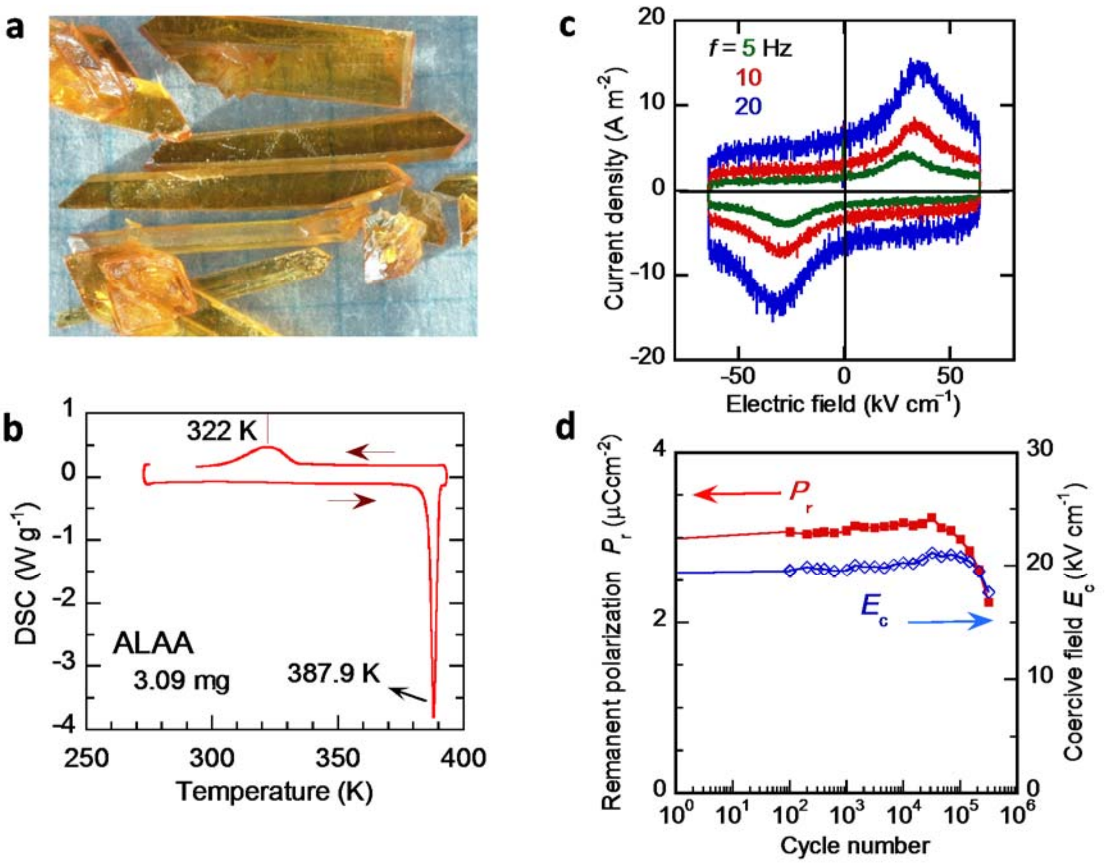

Supplementary Figure 12: Physical properties of ALAA. (**a**) Photograph of the single crystals. (**b**) Differential scanning calorimetry (DSC) of the powder. (**c**) Optimization of *c*-direction polarization. Current density (*J*) versus electric field (*E*) curves at various frequencies for optimized *P*–*E* hysteresis loops. Continuous rectangular-pulse voltages (f = 100 Hz, E^{puls} = 60 kV cm^{-1}, 3 × 10^4 cycles) were applied for optimization. (**d**) Evolution of P_r and E_c measured with a triangular waveform voltage (f = 10 Hz, E^{max} = 60 kV cm^{-1}) in the endurance measurement modes.

Supplementary Table 1: Symmetric and molecular structural properties of PTM ferroelectric crystals.

Compound	Z [Z']	Space [point] group (#)		Polarization	Molecular property	
		Ferroelectric	(Hypothetical) paraelectric		No of H^+	Pseudo-sym
**1**. CRCA	4 [1]	$Pca2_1$ [mm2] (#29)	$Pcam$ [mmm] (#57)	$\|\|c$	2	σ_v
**2**. PhMDA	4 [1]	$Pna2_1$ [mm2] (#33)	$Pnab$ [mmm] (#60)	$\|\|c$	1	C_2
**3**. HPLN	8 [4]	Pn [m] (#7)	$P2_1/n$ [2/m] (#14)	$\perp b$	1	i on dimer
**4**. CBDC	4 [1]	Cc [m] (#9)	$C2/c$ [2/m] (#15)	$\perp b$	2	C_2
**5**. MBI	8 [4]	Pn [m] (#7)	$P4_2/n$ [4/m] (#86)	$\perp b$	1	C_2 on tetrad
**6**. DC-MBI	4 [1]	$Pca2_1$ [mm2] (#29)	$Pcam$ [mmm] (#57)	$\|\|c$	1	σ_v
**7**. ALAA	8 [1]	$Iba2$ [mm2] (#45)	$Ibca$ [mmm] (#73)	$\|\|c$	1	C_2

Supplementary Table 2: Hydrogen-bond lengths (Å) and angles (°) of energetically optimized H positions.

Compound		Distance			Angle
	D – H ··· A	D ··· A	D – H	H ··· A	D – H ··· A
**1**. CRCA	O1–H1···O5 ^{a}	2.614	1.03	1.60	167.48
	O2–H2···O4 ^{a}	2.633	1.03	1.61	171.81
**2**. PhMDA	O1–H1···O2 ^{a}	2.604	1.03	1.57	175.77
**3**. HPLN	O2–H2···O5 ^{a}	2.545	1.05	1.50	176.60
	O4–H10···O7 ^{a}	2.580	1.04	1.54	176.83
	O6–H18···O1 ^{a}	2.586	1.04	1.55	177.40
	O8–H26···O3 ^{a}	2.566	1.05	1.52	176.76
**4**. CBDC	O2–H5···O3*	2.614	1.03	1.59	173.49
	O4–H6···O1 ^{a}	2.633	1.03	1.61	173.60
**5**. MBI	N1–H1···N4 ^{a}	2.960	1.05	1.91	177.14
	N3–H9···N2	2.861	1.06	1.81	178.53
	N5–H17···N8 ^{a}	2.965	1.05	1.92	176.20
	N7–H25···N6	2.859	1.06	1.80	178.79
**6**. DC-MBI	N2–H3···N1 ^{a}	2.977	1.04	2.02	151.53
**7**. ALAA	N2–H9···N1 ^{a}	2.965	1.05	1.92	172.20

a Atom on symmetry-related neighboring molecule.

* Intramolecular hydrogen bond; otherwise, intermolecular bonds.

Supplementary Table 3: Crystal data and experimental details of ALAA and DBT crystals.

	ALAA	DBT	DBT
Chemical formula	$C_{15}H_{14}N_2$	$C_{15}H_{10}O_2$	$C_{15}H_{10}O_2$
Formula wt.	222.29	222.24	222.24
Temperature (K)	296	300	130
a (Å)	20.165(3)	13.177(9)	12.932(3)
b (Å)	16.110(3)	8.568(6)	8.5868(19)
c (Å)	7.6964(12)	9.654(6)	9.612(2)
α (deg.)	90	90	90
β (deg.)	90	102.743(16)	103.735(8)
γ (deg.)	90	90	90
V (Å^3)	2500.2(7)	1063.0(12)	1036.8(4)
Crystal system	orthorhombic	monoclinic	monoclinic
Space group	*Iba*2 (#45)	*C*2/*c* (#15)	$P2_1/c$ (#14)
ρ_{calc} (g cm^{-3})	1.181	1.389	1.424
Z	8	4	4
Dimensions (mm)	0.42×0.20×0.07	0.25×0.20×0.10	0.25×0.20×0.10
Radiation	MoKα (λ = 0.7108 Å)	Synchrotron (λ = 0.6881 Å)	Synchrotron (λ = 0.6881Å)
$2\theta_{max}$ (deg.)	55	70.0	100
R_{int}	0.028	0.027	0.040
Reflection used ($2\sigma(I) < I$)	1504	1236	3189
No. of variables	159	101	194
R	0.038	0.050	0.060
R_w	0.102	0.041	0.060
GOF	1.08	1.05	1.12

Supplementary Note

P–E hysteresis data

The DBT crystal exhibited $P = 0$ at zero field without the opening of a loop. The slightly S-shaped curve is accompanied by two positive and negative peak couples at $E \sim \pm 80$ kV cm^{-1} in the corresponding *J–E* curve (Supplementary Fig. 7c). These features signified antiferroelectricity. We also checked the possibility of strong pinning ferroelectric DWs under strong ac field cycling. The *P–E* hysteresis curve did not change at all even after applying 5×10^3 cycles of a bipolar rectangular-pulse voltage (f = 100 Hz; E^{puls} = 100 kV cm^{-1}). Thermal treatment did not open the loop, indicating the absence of a ferroelectric state even at higher temperatures at least up to 110 °C.

Thermal Analysis

The high-sensitive thermal analysis of DBT (Supplementary Fig. 12b) was performed using a differential scanning calorimeter (Hitachi High-Tech Science Corporation DSC7000X). The temperature was calibrated using the melting point of indium (429.8 K). The sample of 3.09 mg was encapsulated in an aluminum pan and heated at a rate of 5 K min^{-1}. The sample melted at 387.9 K with latent heat of 27.8 kJ mol^{-1}. The ferroelectric state is stable at least up to the melting point, below which the signature of the phase transition was absent until room temperature.

X-ray crystallographic data

Supplementary Table 2 summarizes the crystal data and experimental details for ALAA and DBT. Cambridge Crystallographic Data Center as supplementary publications no. CCDC-1498921-1498923

Synchrotron X-ray diffraction studies of DBT. For the analysis of DBT at room temperature, we referred to earlier studies by Mochida *et al.*[1], which reported a crystal structure with polar crystal symmetry (monoclinic, space group *Cc*). Because this report was found to be inconsistent with our findings of pseudo-symmetry and silent ferroelectricity, we have made structural reassessments using synchrotron X-ray studies. X-ray diffraction experiments at T = 300 and 130 K were carried out with a Rigaku DSC imaging plate diffractometer and synchrotron radiation (λ = 0.68814 Å) on beamline BL-8B at the Photon Factory (PF), High-Energy Accelerator Research Organization (KEK). The X-ray beam was monochromatized by a Si double-crystal monochromator and focused by a bent cylindrical mirror made of a Si crystal coated with Rh. The reflection intensity data were collected with the use of the Rapid-AUTO software package (Rigaku Corp.) and analyzed with the CrystalStructure crystallographic software packages (Molecular Structure Corp. and Rigaku Corp.). Final refinements were done with anisotropic atomic displacement parameters for the non-hydrogen atoms.

At room temperature (T = 300 K), the routine procedure for data processing suggested a C-centered monoclinic lattice. The structural parameters were refined by assuming an acentric Cc and its higher $C2/c$ symmetry. The asymmetric unit of the latter structure comprises a half molecular unit on a twofold rotation axis, and the β-diketone enol unit then exhibited a proton-disordered configuration of averaged symmetry between O=C–C=C–OH and its conjugate HO–C=C–C=O forms. In the former acentric structure, one DBT molecule is crystallographically independent, but the geometry of the β-diketone enol unit revealed an inaccurate bond-alternation geometry. We could not find any validity for the choice of the acentric structure, considering also the lack of improvement in reliability factors compared with the centric structure. Instead, we noticed the appearance of many satellite (hkl) reflections ($h + k$ = odd), which indicated the symmetry reduction from a C-centered to primitive lattice (Supplementary Fig. 8a). The satellite reflections were weaker in intensity by 2–3 orders of magnitude than the typical intense fundamental reflections. Their weak intensity manifested small structural modifications from the $C2/c$ symmetry into a primitive lattice superstructure but prevented us from obtaining a satisfactory structural analysis.

For the full crystal structure analysis in the primitive cell, we examined the data collection using the synchrotron X-ray source at low temperature T = 130 K to develop the degree of superstructural ordering. As expected, the satellite (hkl) reflections ($h + k$ = odd) were only 1–2 orders of magnitude smaller in intensity than the typical intense fundamental reflections (Supplementary Fig. 8a). The analysis with the centric space group $P2_1/c$ satisfactorily determined the location of protons and the β-diketone enol bond geometry. The intermolecular O–H···O bonds construct a zigzag chain parallel to the crystal c-direction (Supplementary Fig. 8c). Because the hydrogen bonds are approximately normal to this chain direction, the polarity of the chain comes from the switchable π-bond dipoles of PTM in analogy with the case of HPLN and ALAA. Each chain polarity is antiparallel to that of the neighboring polarity along the a-direction (Supplementary Fig. 8b). In summary, the observed antiferroelectric behavior is consistent with the antiparallel arrangement and highly polarizable nature of the polar chains, as is evident from the small structural difference between the proton-disordered ($C2/c$) and antiparallel-ordered ($P2_1/c$) configurations.

Supplementary References

1. Mochida, T., Izuoka, A., Sato, N. & Sugawara, T. Decarboxylation of crystalline 2-carboxy-3-hydroxydibenzo[a,c]tropone followed by solid-state crystallization. *Chem. Phys. Lett.* **210,** 389–392 (1993).
